# *Actinomadura welshii sp. nov.*, a New Mycetoma Agent in Mexico

**DOI:** 10.1371/journal.pntd.0013016

**Published:** 2025-04-11

**Authors:** Lucio Vera-Cabrera, Carmen A. Molina-Torres, Adele E. Crane, Mayra G. Cantú-Alvarez, Mario A. Aguilera-Valenciano, Anabel Gallardo-Rocha, Wendy G. Escalante-Fuentes, Jorge Ocampo-Candiani, Charlotte Avanzi

**Affiliations:** 1 Laboratorio Interdisciplinario de Investigación Dermatológica, Servicio de Dermatología, Hospital Universitario “José Eleuterio González”, Nuevo León, México; 2 Department of Microbiology, Immunology and Pathology, Colorado State University, Fort Collins, Colorado, United States of America; University of Khartoum, SUDAN

## Abstract

*Actinomadura* isolates obtained from seven human mycetoma cases in Mexico were characterized using nucleotide sequence analysis of a portion of the small subunit ribosomal RNA gene. Most isolates were identified as *Actinomadura madurae.* However, one isolate, LIID-AQ337, showed inconclusive results. To determine its identity, genomic DNA from LIID-AQ337 was subjected to whole-genome sequencing using both short- and long-read sequencing, resulting in a genome of 8,163,638-bp spread in 22 contigs. Comparative analysis against currently available genomes of other *Actinomadura* species suggested that LIID-AQ337 belongs to a new *Actinomadura* species. We propose to name it *Actinomadura welshii sp. nov* in honor of Oliverio Welsh, a Mexican dermatologist dedicated to mycetoma studies in Mexico.

## Author summary

Mycetoma is still a neglected infectious disease present in Mexico. The diagnosis and identification of the etiological agents have relied on the use of conventional methods for decades. The application of modern DNA-based molecular biology methods has allowed us to identify the etiological agents with similar microscopic and histopathologic appearances. Since mycetoma is caused by many fungal and bacterial agents, they may share certain biological characteristics that enable them to grow in the human host. Comparative genomic analysis may help us study these otherwise cryptic organisms. In this work, we identify a new *Actinomadura* species from a human mycetoma case and analyze its genomic composition through whole-genome analysis.

## Introduction

Mycetoma is a chronic granulomatous infection of the skin, especially in lower extremities, and is characterized by painless subcutaneous masses progressing to open infection discharging grains [1]. Globally, the disease is equally caused by fungal pathogens (eumycetoma) or bacterial pathogens (actinomycetoma). The etiological agents live normally as saprophytes in the soil but may penetrate the skin through trauma and slowly disseminate to the contiguous subcutaneous tissue [1]. Common fungal culprits include *Madurella mycetomatis*, *Falciformispora* (*Leptosphaeria*) *senegalensis*, *Scedosporium apiospermum*, and others (2). Actinomycetoma is primarily caused by aerobic actinomycetes including the genera *Nocardia*, *Actinomadura* and *Streptomyces* [2].

In Mexico, 96% of the cases are attributed to bacteria, with *Nocardia brasiliensis* being the most prevalent (65%), followed by *Actinomadura madurae* (8%) [3]. *A. madurae* belong to the *Thermonosporaceae* family and can be identified by its specific histopathological features: large, white-yellowish grains (typically measuring between 0.8 to 2 mm), that stain strongly basophilic with hematoxylin and eosin (H&E) [1]. In culture, it produces glabrous, waxy, irregular colonies, that test positive for casein hydrolysis [4]. Confirmation of actinomycetes’ identity can be achieved through PCR using primers targeting hypervariable regions or the complete gene encoding the small subunit ribosomal RNA (16S rRNA), followed by nucleotide sequence analysis [5].

Recent genomic analysis of *Actinomadura* and *Streptomyces* species using molecular methods revealed more diversity than previously identified in both genera [6]. In this study, we subjected seven *Actinomadura* isolates from human mycetoma cases from Mexico to nucleotide sequence analysis of the 16S rRNA. Further analysis using whole-genome sequencing was necessary for one isolate, LIID-AQ337, which did not yield conclusive 16S rRNA characterization and led to the identification of a new *Actinomadura* species named *Actinomadura welshii sp. nov.*

## Methods

### Ethics Statement

The present work is part of the project No. DE24–00015, which has been approved by the Comité de Ética en Investigación (CONBIOETICA 19-CEI-001–20160404) of the Facultad de Medicina, Universidad Autónoma de Nuevo León. According to this protocol, verbal consent was obtained from the patients to obtain the clinical samples.

### Bacterial Isolation, Culturing and Characterization

The patients attended the Servicio de Dermatología at the Hospital Universitario “José E. González,” Universidad Autónoma de Nuevo León. Skin tissue, grains, or pus material were obtained through surgical biopsy or lesion puncture and seeded on Sabouraud Dextrose agar, Mycobiotic agar, or Brain Heart Infusion (BHI) agar enriched with 5% blood for culture isolation at 37°C for up to 20 days. Growth rate and colony morphology were recorded and Gram-staining was prepared from each isolate using Kinyoun staining [7]. Hydrolysis tests for tyrosine, casein, hypoxanthine, and xanthine were also performed in duplicate as routinely conducted in our facility for Actinomycete identification [4]. Samples of all cultured isolates were preserved at -80°C in 20% skimmed milk for further analysis.

### Histopathological Analysis

Deep surgical biopsies were collected and stored in 4% neutral buffer formalin before embedding in paraffin. Sections of 3–5 μm obtained using a rotary microtome (Leica, Germany) were stained with haematoxylin and eosin (H&E), Periodic Acid Schiff (PAS) and Grocott methenamine silver (GMS) stains and examined for the presence of characteristic grains.

### Disk diffusion susceptibility test

Antimicrobial susceptibility tests were conducted as previously described [8]. In brief, all seven *Actinomadura* cultures and the wild type ATCC25292 strain were grown on BHI 37°C with agitation until reaching growth phase (optical density 0.4-0.6). The bacterial mass was collected by centrifugation and ground using a Potter-Evelham device (ThermoFisher Scientific, New Hampton, NH, USA). The suspension was then centrifuged at 100xg, and the supernatant was adjusted to McFarland tube number 1 (ThermoFisher Scientific, New Hampton, NH, USA). This suspension was plated onto Mueller-Hinton agar (Oxoid), and antimicrobial susceptibility disks (Oxoid) were impregnated with the following compound concentrations: amikacin (30 μg), amoxicillin/clavulanic acid (30 μg), linezolid (30 μg), moxifloxacin (5 μg), levofloxacin (5 μg), netilmycin (30 μg), ticarcillin/clavulanic acid (85 μg), sulfamethoxazole/trimethoprim (SXT) 1:19 (25 μg), imipenem (10 μg), and rifampicin (5 μg). The disks were placed on the plates immediately after inoculation and incubated at 37°C for five days before measuring the zones of inhibition in millimeters. A heat map was prepared with the inhibition halos measurement using pheatmap and vidiris in R studio v 2024.09.1+394.

### DNA Isolation and 16S rRNA amplification

The bacterial cultures of isolates LIID-AT157 and LIID-AQ337 were expanded from a pre-cultured stock in 5mL of BHI broth at 37°C with shaking at 110 rpm for 72 hours until reaching growth phase (optical density 0.4-0.6). DNA extraction was performed using the cetyltrimethylammonium bromide (CTAB) method [9]. Bacterial identity was determined through substrate hydrolysis pattern analysis [4] and through molecular means by amplifying a fragment of the 16S rRNA gene using primers NOC3 and NOC4 [5]. These primers were designed to amplify a 235-bp DNA region from various pathogenic actinomycetes. The sequence of the purified PCR product was determined on an ABI PRISM DNA sequencer using the Prism Dye Terminator Cycle sequencing kit (Applied Biosystems, Foster City, Calif.). 16S rRNA sequences were compared to the available sequences of LIID-AO173, LIID-1J290, LIID-AN189, LIID-AO236 previously sequenced in our laboratory [10,11] and MRC0005, an *Actinomadura* strain sequenced from Sudan [6]. The alignment was visualized using Jalview v. 2.11.3 [12] and Geneious Prime v2024-0–7.

### DNA isolation, genome sequencing, and assembly

The bacterial culture of isolate LIID-AQ337 was expanded in 5 mL of BHI broth at 37°C with shaking at 110 rpm for 72 hours until reaching growth phase (optical density 0.4-0.6), and DNA extraction was performed using the Qiagen Genomic-tip 20/G as per manufacturer recommendation for gram-positive bacteria. Fragment quality and DNA concentration were checked by Tape Station (Agilent) and Qubit (Thermofisher), respectively.

The library for long-read sequencing was prepared using a ligation sequencing kit (SQK-LSK110, Oxford Nanopore), following manufacturer’s protocols. Sequencing was carried out on one flow cell (Flo-MIN106). For short-read sequencing, 500 ng of DNA was fragmented using a Covaris M220 instrument to 300-bp and a library was prepared using the Kapa HyperPrep kit (Roche) and the KAPA Unique Dual-Indexed Adapter kit (Roche) as per manufacturer recommendations. The quality of the library was determined through the TapeStation (Agilent) and quantified using the Qubit fluorometer. The sample was multiplexed with others and sequencing was carried on a NextSeq 500 instrument using single-end 75 bp sequencing at Colorado State University. Illumina raw reads were adapter- and quality-trimmed with Trimmomatic v0.35 (10). The quality settings were “SLIDINGWINDOW:5:15 MINLEN:40”. For Nanopore sequencing, the resulting reads were basecalled using Guppy v5.0.11 using the fast-base calling model. The min_qscore for read filtering was 8. Draft genome assemblies from Nanopore and Illumina sequences were generated with SPAdes v3.14.0 [13] using the trimmed Illumina reads and Minion reads that passed the filters. The genome was assembled using Mauve [14] using *A. livida* (WGS accession JACHMV01) as a reference. We used the Type (Strain) Genome server [15] to decipher the genome size and G + C content.

### 16S rRNA taxonomy and phylogenetic analysis

To determine whether the isolate LIID-AQ337 represents a novel bacterial species, we first extracted 16S rRNA sequenced from the SPAdes output scaffold (Sup. File). The 16S rRNA sequence of LIID-AQ337 was processed through NCBI BLAST [16], as well as through the EZBioCloud 16S ID tool [17], both of which were set for default parameters. From the top 50 results of each program, we identified 88 unique species and strains with high similarity to LIID-AQ337 (S1 Table). These strains were downloaded from the different platforms, aligned with mafft v7.526 [18], and trimmed with trimAL v1.50 using the “-automated1” parameter [19]. An alignment of 88 strains and 2102 sites including LIID-AQ337 was used to generate a phylogenetic tree in IQ-TREE 2 [20]. The alignment was run with a TIM3+G4 nucleotide substitution model based on ModelFinder results [21]. A bootstrap test of phylogeny was performed using the ultrafast bootstrap function [22] with 1000 replicates. The tree was visualized in iTOL [23]. To confirm topology, a maximum parsimony tree was run in MEGA11 [24] with a bootstrap test of phylogeny with 1000 replicates and a site coverage cutoff of 90%.

### Genome Taxonomy Database (GTDB) Analysis and Average Nucleotide Identity (ANI)

For taxonomic assignment, we used the most recent release of GTDB [25], which integrates genomic data for organisms classified based on average nucleotide identity (ANI) and conserved marker proteins. More specifically, to assign taxonomic labels to our microbial genomes, we employed **GTDBtk** (version v2.4.0) [26]. GTDBtk is a software toolkit designed to apply the GTDB taxonomy framework to bacterial and archaeal genomes. The whole LIID-AQ337 genome was processed through GTDB-Tk classify workflow (“classical_wf”), which compares the query genome against a database of representative GTDB whole genomes, identifies marker genes and calculates ANI-based phylogenetic relationships to assign species and genus-level taxonomy.

## ResultS

### Patients and Clinical isolates of *Actinomadura*

A total of seven *Actinomadura* strains were isolated from patients diagnosed with mycetoma between 2011 and 2022 (Table 1).

**Table 1:** General characteristics of the *Actinomadura* isolates included in this study. They are ordered according to the date of isolation at the Hospital Universitario “José E. González,” Universidad Autónoma de Nuevo León.

Code	Date of isolation	Clinical localization	Molecular identification (16SRNA)	Origin of patient
LIID-AJ290	30/11/11	Mycetoma, right foot	*Actinomadura madurae*	Unknown
LIID-AÑ189	06/07/16	Mycetoma, left foot	*Actinomadura madurae*	Santa Catarina, Nuevo León
LIID-AO173	15/06/17	Mycetoma, right foot	*Actinomadura madurae*	Aramberri, Nuevo León
LIID-AO255	15/09/17	Mycetoma, right hand	*Actinomadura madurae*	Piedras Negras, Coahuila
LIID-AQ337	23/08/19	Mycetoma, left foot	*Actinomadura spp*	San Buenaventura, Coahuila
LIID-AQ397	21/10/19	Mycetoma, left foot	*Actinomadura madurae*	Cadereyta, Nuevo León
LIID-AT157	25/05/22	Mycetoma, left foot	*Actinomadura madurae*	Juárez, Nuevo León

All cases were associated with mycetoma of small size, primarily affecting the feet and not invading beyond the ankle (S1 Fig). One strain, LIID-AJ290, isolated in 2011, was previously characterized as *A. madurae* by our group using genome sequencing and was used as control in our experiments [11]. Four of the cases were previously identified as *A. madurae* using morphological, growth, histopathology and 16s rRNA sequencing by our group [10]. Two cases (LIID-AQ337 and LIID-AT157) were novel strains characterized in this study. Patients’ history, symptoms and disease evolution are described in S1 File. Microscopic examination of the pus showed about 1mm typical white grains (S2 Fig). Slow growing, waxy, glabrous, white-pink colonies characteristic of *Actinomadura spp* were observed after 5–20 days of culture on Saboureaud agar at 37°C. Microscopically, all samples stained negative on acid-fast staining using the Kinyoun method, suggesting the presence of *Actinomadura* bacteria. All samples were negative for casein, tyrosin, xanthine, hypoxantine, and urea hydrolysis tests.

### Antimicrobial susceptibility testing

**Fig 1:**
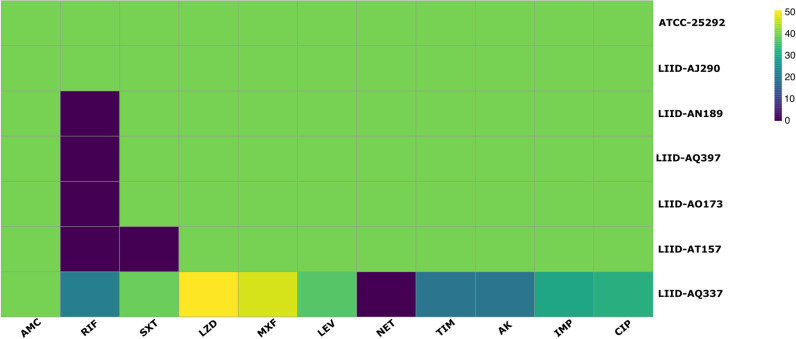
Antimicrobial susceptibility patterns of the *Actinomadura* species tested in this study. The graph was prepared using the packages pheatmap and vidiris in R studio v 2024.09.1+394. Inhibition zones (mm) are depicted from less active (purple) to more susceptible (green). The following antimicrobials were used: Linezolid (LZD), Moxifloxacin (MXF), Levofloxacin (LEV), Netilmycin (NET), Ticarcillin-Clavulanate (TIM), Trimethoprim-sulfamethoxazole (SXT), Amikacin (AK), Imipenem (IMP), Rifampicin (RD), Amoxicillin-Clavulanate (AMC) and ciprofloxacin (CIP).

Generally, all *Actinomadura* isolates tested were susceptible to the first line drugs (amoxicillin/clavulanic acid and SXT) and resistant to rifampicin except for *Actinomadura madurae* LIID-AT157 which was resistant to SXT and *Actinomadura madurae* LIID-AJ290, which was sensitive to rifampicin (Fig 1). In contrast to these results, previously published strains of *A. madurae* from Yemen and Sudan (6) were completely resistant to SXT.

*Actinomadura* LIID-AQ337 showed resistance to netilmicin and a smaller inhibition halo to ticarcillin-clavulanate, amikacin, imipenem, and rifampicin (Fig 1). Treatment for 6 months with SXT and amoxicillin/clavulanate lead to complete remission of the lesions in the patient infected with *Actinomadura* LIID-AQ337 strain (S1 Fig).

### Species identification

To confirm the morphologic characterization of the isolates, the 16S rRNA sequence obtained by PCR-sequencing (NOC3-NOC4) was used for species identification (S3 Fig). Five isolates show a 100% homology to *Actinomadura madurae* strain DSM43067, suggesting that all five isolates are *A. madurae*. Interestingly, one isolate, LIID-AQ337, shows divergence. The partial 16S rRNA sequence (S_16S_AQ337) was then aligned against the GenBank database using the tool BLAST. The closest hit is an uncharacterized *Actinomadura* species, strain CC0560 (99% query cover and 98.23% identity). The closest characterized species was *A. livida* (99% query cover and 97.84% identity), suggesting that LIID-AQ337 might belong to a yet uncharacterized *Actinomadura* species.

### Genome analysis of LIID-AQ337

To further characterize this new species, we performed *de novo* assembly of short- and long-read sequences generated from LIID-AQ337 isolate. The SPAde assembly resulted in a scaffold of 145 contigs larger than 200-bp including 25 contigs larger that 600-bp, 16 larger than 100,000, with the largest of 1,248,311 bp. According to TYGS analysis [15], *Actinomadura* LIID-AQ337 has a G+C content of 72.51% and a genome size of 8,163,638-bp.

### 16S rRNA analysis

To further confirm that *Actinomadura* LIID-AQ337 is a new species, we isolated the 16S rRNA from the whole-genome sequence (S2 File). The EZBioCloud 16S tool yielded 16S sequences from 47 members of the *Actinomadura* genus, two *Sprillospora* members, and one *Thermomonospora* species. *Actinomadura sputi* had the highest pairwise similarity at 98.056 and a mismatch score of 28/1440 total nucleotides. The BLAST results showed the same ratio of *Actinomadura*, *Sprillospora*, and *Thermomonospora* members represented. The highest alignment score (Max Score) is determined from the sum of rewards for matched nucleotides, mismatches and gaps. *Actinomadura sp.* CC0580 had the highest Max Score and had a Max Score/Percent identity of 2662/98.23%. The highest percent identity was *Actinomadura sp* strain 6K520 at 98.26%.

**Fig 2:**
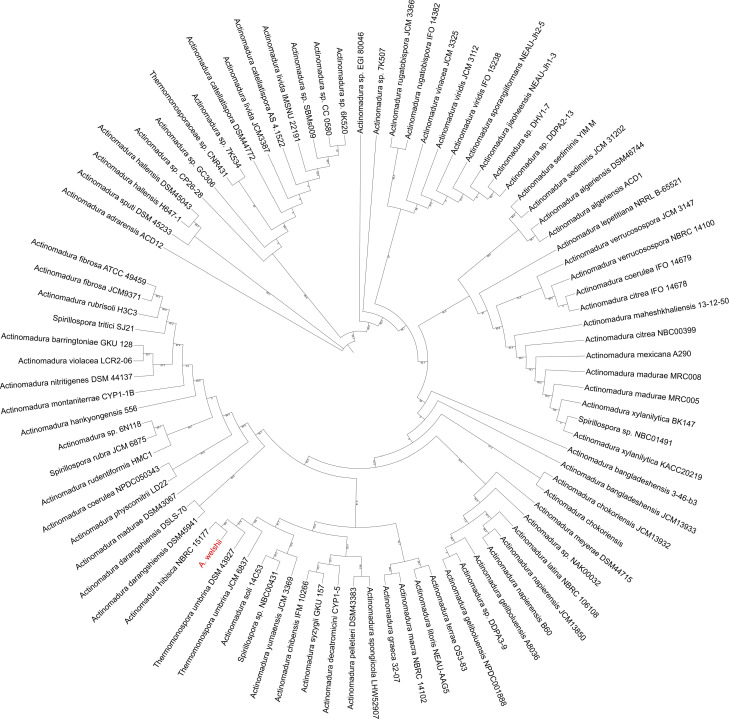
The 16S rRNA phylogeny of closest species of LIID-AQ337. The tree shows the placement of 88 isolates, including LIID-AQ337 (in red), with related 16S rRNA sequences from the ezBioCloud and NCBI databases. Inferred using iqtree2 and the TIM3+G4 model. The closest species is *Actinomadura hibisca* NBRC15177 with a bootstrap 100.

A list of comparative data was generated from these results for a total of 87 unique strains and species to be used in 16S rRNA and ANI analysis. For the 16S rRNA phylogeny, an alignment of 88 strains and 2102 sites including LIID-AQ337 was generated and analyzed in IQ-TREE 2 [19] with a TIM3+G4 nucleotide substitution model. The resulting tree (Fig 2) placed LIID-AQ337 with *Actinomadura hibisca* strain NBRC15177 and within a larger clade containing *Thermomonospora umbrina* DSM 43927 and JCM 6837 strains. The maximum parsimony tree also supported *A. hibisca* as the closest relative, but the clade contained *A. coerulea*, *S. rubra* and *S. tritici* (S4 Fig).

### GTDB analysis

The LIID-AQ337 genome was classified under the phylum Actinomycetota, class Actinomycetes, order Streptosporangiales, family Streptosporangiaceae and genus *Spirillospora*. However, LIID-AQ337 was unable to be assigned a close relative species or strain through the GTDB-Tk classify workflow as the results fell outside of the pre-defined ANI radius (95% ANI circumscription). The ANI analysis yielded 47 strains ranging from 82.54% (*Spirillospora macra*) to the closest species with an ANI of 89.87% (95% confidence interval) for *Spirillospora livida* (*Actinomadura livida*) (S2 Table).

We obtained a 91.26% Multi-Sequence Alignment score suggesting that most of the core marker genes matched GTDB references, meaning our genome is well-sequenced and correctly formatted. Furthermore, we obtained a high “red value” (0.98) suggesting that the classification is reliable, even though the genome was not assigned to an existing species. These GTDB results indicate that LIID-AQ337 might belong to a new species. We propose to name this new species *Actinomadura welshii sp. nov.* in honor of Oliverio Welsh, a Mexican dermatologist who dedicated his career to the study of mycetoma in Mexico.

The GTDB’s standardized bacterial taxonomy, utilized by GTDBtk, combines the genus *Spirillospora* with several species that are traditionally classified under the genus *Actinomadura* in NCBI and LPSN (Approved Lists) [6]. In this merged taxonomy, all members are now classified as *Spirillospora*, with *Spirillospora albus* being the type species. This also includes *Actinomadura madurae*, which is renamed *Spirillospora madurae* in the GTDB. All isolates identified as belonging to the *Spirillospora* genus by GTDBtk were confirmed to be *Spirillospora madurae* (formerly *Actinomadura madurae*). For consistency, we chose to retain the previously established genus name *Actinomadura madurae* when referring to the taxonomic classification of these isolates throughout this manuscript, as it remains the valid species name in LPSN (Approved Lists) and NCBI and is more widely recognized within the actinomycetoma research community. This was also employed in the latest comparative genomic study performed on *Actinomadura* strains [6].

## Discussion

The genus *Actinomadura* comprises 78 species that have been validly published, with *Actinomadura madurae* being the type species (4). Here, we report the identification of a new *Actinomadura* species called *A. welshii sp. nov.* as a causative agent of human mycetoma in Mexico.

While many species have been reported as human pathogens, only a few, such as *Actinomadura madurae*, *Actinomadura mexicana*, and *Actinomadura pelletieri* [1,2,27] have been reported in human mycetoma cases. In Mexico, most cases have been attributed to *A. madurae* [3]. However, identification is typically based on colony morphology, histopathological characteristics of the grains, and the hydrolysis of various compounds [1,4]. Molecular DNA-based techniques have revealed that actinomycetes previously identified as “*spp*” may actually represent different species [6].

*Actinomadura* LIID-AQ337 exhibited clinical, morphological, and histopathological characteristics resembling those of *A. madurae.* However, its 16S rRNA nucleotide sequence was notably different. Upon analyzing the complete 16S rRNA sequence, strain LIID-AQ337 appears to be most closely related to the type strain of *A. hibisca* NBRC15177. The ANI analysis performed on the whole genome of LIID-AQ337 confirmed its classification within the genus *Actinomadura*. Moreover, the inability to assign this genome to a specific species or strain using the GTDB-Tk classify workflow highlights its genetic divergence from known taxa and confirms that LIID-AQ337 belongs to a new species. The pre-defined ANI radius of 95% serves as a standard for species delineation, and our results indicate that LIID-AQ337 falls outside this threshold. The ANI analysis revealed genetic similarities with 47 strains, with the closest match being *Spirillospora livida* (also known as *Actinomadura livida*) at 89.87% ANI, while *Spirillospora macra* exhibited the lowest similarity at 82.54% ANI.

Interestingly, both closest species, *A. hibisca* [28] and *A. livida* [29] are mostly found in soil samples and to a less extent in plants, water or animals. Our strain was isolated from a human mycetoma case in San Buenaventura, Coahuila, a semi-desertic region in Mexico. Mycetoma-producing actinomycetes inhabit the soil and enter the human body through minor trauma, such as thorn or wood splinter injuries [1]. Once inside the skin, they slowly disseminate and destroy contiguous tissues, including muscle, fascia, or bones. Lower extremities are the most commonly affected areas. However, people living in these rural areas are continuously exposed to minor traumas, and there are only a few cases of mycetoma. Microorganisms that successfully invade host tissues may possess special characteristics. More sequencing of human infecting *Actinomadura* and comparison with environmental *Actinomadura* might lead to the identification of specific virulence genes, metabolites and possible new targets for antimicrobials. Indeed, actinomycetes includes a wide range of bacteria that have been studied by their production of multiple metabolites with diverse biological functions, including anti-cytostatic, immunosuppressive, antimicrobial, and antiviral activities [30,31]. Although most of these metabolite producing microorganisms belong to the *Streptomycetaceae* family, *Thermomonosporaceae* species have also been found to be important in this area. Even the pathogenic species *A. madurae* has been found to produce several compounds, including the potent anti-tumor maduropeptin [32]. Although most of these compounds have come from terrestrial or marine *Actinomadura* isolates, isolates from human infections may also be a source of these metabolites. In an actinomycetoma case due to *N. brasiliensis,* an immunosuppressive status has been reported [33]. It is possible that actinomycetes may produce *in vivo* substances that may modulate the immune system, helping these microorganisms to establish the infection, although this hypothesis has yet to be tested.

The pattern of antimicrobial susceptibility of *A. welshii* suggest that the new species is sensitive to the first line drug SXT and amoxicillin-clavulanate. However, it is partially or fully resistant to most of the other drugs tested in our assay suggesting that the range of drugs available to treat mycetoma infection with *A. welshii* might be limited in case of resistance to first line drugs. Interestingly, when comparing the SXT susceptibility pattern of our Mexican *A. madurae*, with those previously reported, we observed a particular resistance to SXT in African isolates. This is of concern as SXT is one of the first line drugs. Systematic analysis of the genes involved in anti-folate resistance might help predict this susceptibility.

In this study, we have described the molecular, biochemical, and susceptibility characteristics of *Actinomadura* LIID-AQ337. Furthermore, we have compared these findings with the molecular and genomic characteristics of other *Actinomadura* species. In summary, LIID-AQ337 exhibits distinct features compared to its closest relatives, suggesting that it should be recognized as a new species. As such, we propose the name *Actinomadura welshii sp. nov.*

### Protologue

Description of *Actinomadura welshii sp. nov.*

*Actinomadura welshii* (wel-sh’ii. NL gen. masc. n. welshii of Welsh, was named in honor of Oliverio Welsh, a Mexican dermatologist, for his vast contributions to the therapy and epidemiology of actinomycetoma)

A non-motile, non acid-fast, filamentous Gram-positive and slow-growing bacterium. Growth on solid media requires culturing for >7 days at temperatures between 31 and 37 °C, optimally at 35 °C, producing glabrous, waxy, membranous, cream-coloured colonies of 1–2 mm in diameter on Brain Heart Infusion agar plates. It does not hydrolyze casein, tyrosine, xanthine, hypoxantine or urea. It is sensitive to linezolid, ciprofloxacin, moxifloxacin, levofloxacin, trimethoprim-sulfamethoxazole, imipenem, and amoxicillin-clavulanate. Resistant to netilmicin, ticarcillin-clavulanate, amikacin, and rifampicin.

The genome of type strain LIID-AQ337 comprises 8,163,638-bp spread in 22 contigs in silico, and the G+C content is 72.51%. The whole-genome sequence and the 16S rRNA gene sequence are deposited in the NCBI GenBank database under the BioProject ID PRJNA1232214 and PRJNA1046528. The strain of *Actinomadura welshii* LIID-AQ337 has been deposited to the U.S. Department of Agriculture Agricultural Research Service (ARS) Culture Collection under the number: NRRL B-65740.

## Supporting information

S1 File Patients’ history. (DOCX)

S2 File 16S rRNA sequence of LIID AQ-337. (DOCX)

S1 Table List of the 88 unique species and strains with high 16S rRNA similarity to LIID-AQ337 from NCBI BLAST AND EZBioCloud. (DOCX)

S2 Table Pairwise genome comparison of *LIID-AQ337* with the species of the GTDB database. (DOCX)

S1 Fig Clinical evolution of three of the mycetoma cases included in this study after therapy. A, A. madurae LIID-AQ397; B, seven months after therapy with SXT alone. C Actinomadura LIID-AQ337; D, seven months after therapy with SXT plus amoxicillin/clavulanate. E, A. madurae LIID-AT157, F, decrease of lesions after therapy with 10 months of SXT and four cycles of amikacin.
(DOCX)

S2 Fig Microscopic examination. In A we show the presence of granules > 1 mm size (10x) in the pus of patient LIID-AT157. In B we show a 40x magnification to show the presence of filaments of less 1 μm diameter. In C we show the presence of typical large basophilic grains in an H&E stained tissue. Bar represents 200 μm.
(DOCX)

S3 Fig Nucleotide sequence alignment of the 16S rRNA amplicons (NOC3-NOC4) derived from the *Actinomadura* isolates from this study and DSM43067 as reference. The alignment was done using Geneious Prime v2024-0–7. This fragment aligned with the nucleotide sequence (positions 3058936–3058745) of the genome sequence of A. madurae DSM43067 (GenBank accession no. X97889) encoding for the 16S ribosomal RNA product.
(DOCX)

S4 Fig Maximum parsimony tree of the 16S rRNA phylogeny of Actinomadura showing the placement of LIID-AQ337 (red) with related 16S rRNA sequences from the EZBioCloud and NCBI. Inferred using MEGA11 with a bootstrap test of phylogeny with 1000 replicates and a site coverage cutoff of 90%. The closest species is Actinomadura hibisca NBRC15177 with a bootstrap 100.
(DOCX)
