# Supplementary material for: Actinomadura welshii sp. nov., a New Mycetoma Agent in Mexico
Source: PLoS Negl Trop Dis. 2025 Apr 11;19(4):e0013016. doi: 10.1371/journal.pntd.0013016 (PMC12021271; doi:10.1371/journal.pntd.0013016)
Supplement: S1 File — (DOCX) [file pntd.0013016.s001.docx]

**1.1. CLINICAL CASE LIID AQ-337**

A 74-year-old male patient, originally from the neighboring state of Coahuila, presented to the clinic with dermatosis located on the dorsum of his left foot. The condition was unilateral and asymmetric, characterized by a hyperchromic patch with areas of retraction and eutrophic scars. The patient reported that the condition began six years ago following a fracture, which subsequently evolved into ecchymosis, with the release of purulent material and the development of whitish pimples. He mentioned having been treated previously with trimethoprim/sulfamethoxazole in combination with amoxicillin. A spindle biopsy was performed for histopathological analysis and culture, and an audiometric study revealed chronic bilateral acoustic trauma. Histopathological results indicated an acute and chronic suppurative inflammatory process with abscess formation, with negative Gram, Ziehl-Neelsen, and PAS stains. Culture results showed slow-growing, white, glabrous, and irregular colonies. Sequencing of part of the 16S ribosomal subunit suggested a possible identification of Actinomadura spp., although the result was not conclusive. Antimicrobial therapy was initiated with sulfamethoxazole/trimethoprim (800/160 mg, three times a day) in combination with amoxicillin/clavulanic acid (875/150 mg, twice a day) for two months. The treatment was continued for a total of 8 months, resulting in a 10/10 improvement, with follow-up care focused on the treatment of atrophic scars.

**1.2. CLINICAL CASE LIID AT-157**

A 30-year-old male patient presented to the dermatology clinic with a dermatosis located on his left ankle and foot. The condition was unilateral and asymmetric, characterized by an indurated, polymorphous mass with a hard, stony consistency, accompanied by post-inflammatory papules and macules with blood crusts, indicating a presumably chronic evolution. The patient reported a 4-year history of the condition with no known trauma but mentioned working in orange groves in Tampico, Tamaulipas. He had sought consultations at various clinics, where bacteriological and fungal cultures were performed, all of which yielded negative results. A spindle biopsy was performed, along with the collection of grains for KOH testing and culture, as well as an audiometric evaluation. Histopathological analysis revealed an acute and chronic inflammatory process with granulation tissue, associated with the presence of filamentous bacteria morphologically consistent with actinomycetes. Staining with PAS, Grocott, and Gram was negative, and the patient’s bilateral hearing was found to be normal. The patient was treated with four cycles of sulfamethoxazole/trimethoprim combined with amikacin, and one cycle of TMP/SMX alone, achieving complete remission of the disease within 5 months. Each cycle consisted of trimethoprim (8 mg/kg/day) and sulfamethoxazole (40 mg/kg/day), administered every 8 hours for 5 weeks, along with amikacin (15 mg/kg/day) every 12 hours for 3 weeks, with 2-week intervals between cycles.
