# Supplementary material for: Actinomadura welshii sp. nov., a New Mycetoma Agent in Mexico
Source: PLoS Negl Trop Dis. 2025 Apr 11;19(4):e0013016. doi: 10.1371/journal.pntd.0013016 (PMC12021271; doi:10.1371/journal.pntd.0013016)
Supplement: S2 File — (DOCX) [file pntd.0013016.s002.docx]

>Awelshii_ LIIDAQ337_16S

AAAGGAGGTGATCCAGCCGCACCTTCCGGTACGGCTACCTTGTTACGACTTCGTCCCAATCGCCGGCCCCACCTTCGACCGCTCCCTCCCTTGACGGGTTGGGCCGCGGGCTTCGGGTGTTGCCGACTTTCGTGACGTGACGGGCGGTGTGTACAAGGCCCGGGAACGTATTCACCGCAGCGTTGCTGATCTGCGATTACTAGCGACTCCGACTTCACGAAGTCGAGTTGCAGACTTCGATCCGAACTGAGACCGGCTTTGAGGGATTCGCTCCACCTCACGGTATCGCAGCCCACTGTACCGGCCATTGTAGCATGTTTGCAGCCCAAGACATAAGGGGCATGATGACTTGACGTCATCCCCACCTTCCTCCGAGTTGACCCCGGCGGTCTCCCATGAGTCCCCAACCACCCGAAGGTGTTGCTGGCAACATGGAACGAGGGTTGCGCTCGTTGCGGGACTTAACCCAACATCTCACGACACGAGCTGACGACAGCCATGCACCACCTGTCACCGGCCCAAAAAGGACCCCGCATCTCTGCGGGATTTCCGGCGATGTCAAGCCTTGGTAAGGTTCTTCGCGTTGCGTCGAATTAAGCAACATGCTCCGCCGCTTGTGCGGGCCCCCGTCAATTCCTTTGAGTTTTAGCCTTGCGGCCGTACTCCCCAGGCGGGGCGCTTAATGCGTTAGCTACGGCGCGGAATCCGTGGAAGAACCCCACACCTAGCGCCCAACGTTTACGGCGTGGACTACCAGGGTATCTAATCCTGTTCGCTCCCCACGCTTTCGCTCCTCAGCGTCAGTACAGGCCCAGAGCACCGCCTTCGCCACCGGTGTTCCTCCCGATATCTGCGCATTTCACCGCTACACCGGGAATTCCATGCTCCCCTACCTGCCTCTAGCCTGCCCGTATCCACCGCAGACCCACAGTTAAGCCGTGGGCTTTCACGACAGACGCGACAAACCGCCTACGAGCTCTTTACGCCCAATAATTCCGGACAACGCTTGCGCCCTACGTATTACCGCGGCTGCTGGCACGTAGTTAGCCGGCGCTTCTTCTGCACCTACCGTCACCCCAAGGGGCTTCGTCGATGCTGAAAGAGGTTTACAACCCGAAGGCCGTCATCCCCCACGCGGCGTCGCTGCGTCAGGCTTCCGCCCATTGCGCAATATTCCCCACTGCTGCCTCCCGTAGGAGTCTGGGCCGTGTCTCAGTCCCAGTGTGACCGGTCGCCCTCTCAGGCCGGTTACCCGTCGTCGCCTTGGTAGGCCATCACCCCACCAACAAGCTGATAGGCCGCGAGCCCATCCCCAACCGATAAATCTTTCCACCCGCAGACCATGCGGCCGCGAGTCACATCCGGTATTAGACCCAGTTTCCCAGGCTTATCCCGAAGTCAGGGGCAGGTTGCTCACGTGTTACTCACCCGTTCGCCGCTCGAGTACCCCCGAAGGGGCCTTTCCGCTCGACTTGCATGTGTTAAGCACGCCGCCAGCGTTCGTCCTGAGCCAGGATCAAACTCTCCATTAA
