# Supplementary material for: Actinomadura welshii sp. nov., a New Mycetoma Agent in Mexico
Source: PLoS Negl Trop Dis. 2025 Apr 11;19(4):e0013016. doi: 10.1371/journal.pntd.0013016 (PMC12021271; doi:10.1371/journal.pntd.0013016)
Supplement: S1 Table — (DOCX) [file pntd.0013016.s003.docx]

**S1 Table: List of the 88 unique species and strains with high 16S rRNA similarity to LIID-AQ337 from NCBI BLAST AND EZBioCloud**

| **Name** | **Strain** | **Full name** | **NCBI Accession** | **Assembly Level** | **Taxonomy** |  |
| --- | --- | --- | --- | --- | --- | --- |
| *Actinomadura adrarensis* | ACD12 | *Actinomadura adrarensis* ACD12 | KU356942 | contig | Bacteria;Actinobacteria;Actinomycetia;Streptosporangiales;Thermomonosporaceae;Actinomadura |  |
| *Actinomadura algeriensis* | ACD1 | *Actinomadura algeriensis* ACD1 | KT259320 | contig | Bacteria;Actinobacteria;Actinomycetia;Streptosporangiales;Thermomonosporaceae;Actinomadura |  |
| *Actinomadura algeriensis* | DSM 46744 | *Actinomadura algeriensis* DSM 46744 | JADBDZ01 | contig | Bacteria;Actinobacteria;Actinomycetia;Streptosporangiales;Thermomonosporaceae;Actinomadura |  |
| *Actinomadura bangladeshensis* | 3-46-b3 | *Actinomadura bangladeshensis* 3-46-b3 | AB331652 | contig | Bacteria;Actinobacteria;Actinomycetia;Streptosporangiales;Thermomonosporaceae;Actinomadura |  |
| *Actinomadura bangladeshensis* | JCM 13933 | *Actinomadura bangladeshensis* JCM 13933 | BAAAMX01 | scaffold | Bacteria;Actinobacteria;Actinomycetia;Streptosporangiales;Thermomonosporaceae;Actinomadura |  |
| *Actinomadura barringtoniae* | GKU 128 | *Actinomadura barringtoniae* GKU 128 | KF667497 | contig | Bacteria;Actinobacteria;Actinomycetia;Streptosporangiales;Thermomonosporaceae;Actinomadura |  |
| *Actinomadura catellatispora* | AS 4.1522 | *Actinomadura catellatispora* AS 4.1522 | AF154127 | contig | Bacteria;Actinobacteria;Actinomycetia;Streptosporangiales;Thermomonosporaceae;Actinomadura |  |
| *Actinomadura catellatispora* | DSM44772 | *Actinomadura catellatispora* DSM44772 | JACHMV01 | contig | Bacteria;Actinobacteria;Actinomycetia;Streptosporangiales;Thermomonosporaceae;Actinomadura |  |
| *Actinomadura chibensis* | IFM 10266 | *Actinomadura chibensis* IFM 10266 | AB264086 | contig | Bacteria;Actinobacteria;Actinomycetia;Streptosporangiales;Thermomonosporaceae;Actinomadura |  |
| *Actinomadura chokoriensis* | JCM 13932Ê | *Actinomadura chokoriensis* JCM 13932Ê | BAAAMW01 | scaffold | Bacteria;Actinobacteria;Actinomycetia;Streptosporangiales;Thermomonosporaceae;Actinomadura |  |
| *Actinomadura chokoriensis* | n/a | *Actinomadura chokoriensis* n/a | AB331730 | contig | Bacteria;Actinobacteria;Actinomycetia;Streptosporangiales;Thermomonosporaceae;Actinomadura |  |
| *Actinomadura citrea* | NBC_00399 | *Actinomadura citrea* NBC_00399 | ASM3622626v1 | complete | Bacteria;Actinobacteria;Actinomycetia;Streptosporangiales;Thermomonosporaceae;Actinomadura |  |
| *Actinomadura citrea* | IFO 14678 | *Actinomadura citrea* IFO 14678 | U49001 | contig | Bacteria;Actinobacteria;Actinomycetia;Streptosporangiales;Thermomonosporaceae;Actinomadura |  |
| *Actinomadura coerulea* | NPDC050343 | *Actinomadura coerulea* NPDC050343 | JBFARK01 | scaffold | Bacteria;Actinobacteria;Actinomycetia;Streptosporangiales;Thermomonosporaceae;Actinomadura |  |
| *Actinomadura coerulea* | IFO 14679 | *Actinomadura coerulea* IFO 14679 | U49002 | contig | Bacteria;Actinobacteria;Actinomycetia;Streptosporangiales;Thermomonosporaceae;Actinomadura |  |
| *Actinomadura darangshiensis* | DSM 45941 | *Actinomadura darangshiensis* DSM 45941 | SMKY01 | contig | Bacteria;Actinobacteria;Actinomycetia;Streptosporangiales;Thermomonosporaceae;Actinomadura |  |
| *Actinomadura darangshiensis* | DSLS-70 | *Actinomadura darangshiensis* DSLS-70 | FN646682 | contig | Bacteria;Actinobacteria;Actinomycetia;Streptosporangiales;Thermomonosporaceae;Actinomadura |  |
| *Actinomadura decatromicini* | CYP1-5 | *Actinomadura decatromicini* CYP1-5 | LC516920 | contig | Bacteria;Actinobacteria;Actinomycetia;Streptosporangiales;Thermomonosporaceae;Actinomadura |  |
| *Actinomadura fibrosa* | JCM 9371 | *Actinomadura fibrosa* JCM 9371 | JBHTGP01 | scaffold | Bacteria;Actinobacteria;Actinomycetia;Streptosporangiales;Thermomonosporaceae;Actinomadura |  |
| *Actinomadura fibrosa* | ATCC 49459 | *Actinomadura fibrosa* ATCC 49459 | AF163114 | contig | Bacteria;Actinobacteria;Actinomycetia;Streptosporangiales;Thermomonosporaceae;Actinomadura |  |
| *Actinomadura geliboluensis* | NPDC001888 | *Actinomadura geliboluensis* NPDC001888 | JBIATF01 | scaffold | Bacteria;Actinobacteria;Actinomycetia;Streptosporangiales;Thermomonosporaceae;Actinomadura |  |
| *Actinomadura geliboluensis* | A8036 | *Actinomadura geliboluensis* A8036 | HQ157187 | contig | Bacteria;Actinobacteria;Actinomycetia;Streptosporangiales;Thermomonosporaceae;Actinomadura |  |
| *Actinomadura graeca* | 32-07 | *Actinomadura graeca* 32-07 | CP059572.1 | chromosome | Bacteria;Actinobacteria;Actinomycetia;Streptosporangiales;Thermomonosporaceae;Actinomadura |  |
| *Actinomadura hallensis* | DSM 45043Ê | *Actinomadura hallensis* DSM 45043Ê | VFPO01 | contig | Bacteria;Actinobacteria;Actinomycetia;Streptosporangiales;Thermomonosporaceae;Actinomadura |  |
| *Actinomadura hallensis* | H647-1 | *Actinomadura hallensis* H647-1 | DQ076484 | contig | Bacteria;Actinobacteria;Actinomycetia;Streptosporangiales;Thermomonosporaceae;Actinomadura |  |
| *Actinomadura hankyongensis* | Gsoil 556 | *Actinomadura hankyongensis* Gsoil 556 | KY078843 | contig | Bacteria;Actinobacteria;Actinomycetia;Streptosporangiales;Thermomonosporaceae;Actinomadura |  |
| *Actinomadura hibisca* | NBRC 15177 | *Actinomadura hibisca* NBRC 15177 | BCRO01000158 | contig | Bacteria;Actinobacteria;Actinomycetia;Streptosporangiales;Thermomonosporaceae;Actinomadura |  |
| *Actinomadura jiaoheensis* | NEAU-Jh1-3 | *Actinomadura jiaoheensis* NEAU-Jh1-3 | KM000835 | contig | Bacteria;Actinobacteria;Actinomycetia;Streptosporangiales;Thermomonosporaceae;Actinomadura |  |
| *Actinomadura latina* | NBRC 106108 | *Actinomadura latina* NBRC 106108 | BCQS01000065 | contig | Bacteria;Actinobacteria;Actinomycetia;Streptosporangiales;Thermomonosporaceae;Actinomadura |  |
| *Actinomadura lepetitiana* | NRRL B-65521 | *Actinomadura lepetitiana* NRRL B-65521 | MH061375 | contig | Bacteria;Actinobacteria;Actinomycetia;Streptosporangiales;Thermomonosporaceae;Actinomadura |  |
| *Actinomadura litoris* | NEAU-AAG5 | *Actinomadura litoris* NEAU-AAG5 | MN700217 | contig | Bacteria;Actinobacteria;Actinomycetia;Streptosporangiales;Thermomonosporaceae;Actinomadura |  |
| *Actinomadura livida* | JCM 3387 | *Actinomadura livida* JCM 3387 | BMRO01 | scaffold | Bacteria;Actinobacteria;Actinomycetia;Streptosporangiales;Thermomonosporaceae;Actinomadura |  |
| *Actinomadura livida* | IMSNU 22191 | *Actinomadura livida* IMSNU 22191 | NR_042032.1 | contig | Bacteria;Actinobacteria;Actinomycetia;Streptosporangiales;Thermomonosporaceae;Actinomadura |  |
| *Actinomadura macra* | NBRC 14102 | *Actinomadura macra* NBRC 14102 | BCQT01000047 | contig | Bacteria;Actinobacteria;Actinomycetia;Streptosporangiales;Thermomonosporaceae;Actinomadura |  |
| *Actinomadura madurae* | DSM 43067 | *Actinomadura madurae* DSM 43067 | FOVH01 | scaffold | Bacteria;Actinobacteria;Actinomycetia;Streptosporangiales;Thermomonosporaceae;Actinomadura |  |
| *Actinomadura madurae* | MRC008 | *Actinomadura madurae* MRC008 | CP094265.1 | chromosome | Bacteria;Actinobacteria;Actinomycetia;Streptosporangiales;Thermomonosporaceae;Actinomadura |  |
| *Actinomadura madurae* | MRC005 | *Actinomadura madurae* MRC005 | CP094266.1 | chromosome | Bacteria;Actinobacteria;Actinomycetia;Streptosporangiales;Thermomonosporaceae;Actinomadura |  |
| *Actinomadura maheshkhaliensis* | 13-12-50 | Actinomadura maheshkhaliensis 13-12-50 | AB331731 | contig | Bacteria;Actinobacteria;Actinomycetia;Streptosporangiales;Thermomonosporaceae;Actinomadura |  |
| *Actinomadura mexicana* | A290 | *Actinomadura mexicana* A290 | AF277195 | contig | Bacteria;Actinobacteria;Actinomycetia;Streptosporangiales;Thermomonosporaceae;Actinomadura |  |
| *Actinomadura meyerae* | DSM 44715 | *Actinomadura meyerae* DSM 44715 | FZOR01 | scaffold | Bacteria;Actinobacteria;Actinomycetia;Streptosporangiales;Thermomonosporaceae;Actinomadura |  |
| *Actinomadura montaniterrae* | CYP1-1B | *Actinomadura montaniterrae* CYP1-1B | LC126428 | contig | Bacteria;Actinobacteria;Actinomycetia;Streptosporangiales;Thermomonosporaceae;Actinomadura |  |
| *Actinomadura napierensis* | JCM 13850 | *Actinomadura napierensis* JCM 13850 | BAAAMR01 | scaffold | Bacteria;Actinobacteria;Actinomycetia;Streptosporangiales;Thermomonosporaceae;Actinomadura |  |
| *Actinomadura napierensis* | B60 | *Actinomadura napierensis* B60 | AY568292 | contig | Bacteria;Actinobacteria;Actinomycetia;Streptosporangiales;Thermomonosporaceae;Actinomadura |  |
| *Actinomadura nitritigenes* | DSM 44137 | *Actinomadura nitritigenes* DSM 44137 | AY035999 | contig | Bacteria;Actinobacteria;Actinomycetia;Streptosporangiales;Thermomonosporaceae;Actinomadura |  |
| *Actinomadura pelletieri* | DSM 43383 | *Actinomadura pelletieri* DSM 43383 | RBWU01 | scaffold | Bacteria;Actinobacteria;Actinomycetia;Streptosporangiales;Thermomonosporaceae;Actinomadura |  |
| *Actinomadura physcomitrii* | LD22 | *Actinomadura physcomitrii* LD22 | MH715905 | contig | Bacteria;Actinobacteria;Actinomycetia;Streptosporangiales;Thermomonosporaceae;Actinomadura |  |
| *Actinomadura rubrisoli* | H3C3 | *Actinomadura rubrisoli* H3C3 | MG211703 | contig | Bacteria;Actinobacteria;Actinomycetia;Streptosporangiales;Thermomonosporaceae;Actinomadura |  |
| *Actinomadura rudentiformis* | HMC1 | *Actinomadura rudentiformis* HMC1 | DQ285420 | contig | Bacteria;Actinobacteria;Actinomycetia;Streptosporangiales;Thermomonosporaceae;Actinomadura |  |
| *Actinomadura rugatobispora* | IFO 14382 | *Actinomadura rugatobispora* IFO 14382 | U49010 | contig | Bacteria;Actinobacteria;Actinomycetia;Streptosporangiales;Thermomonosporaceae;Actinomadura |  |
| *Actinomadura rugatobispora* | JCM 3366 | *Actinomadura rugatobispora* JCM 3366 | AP029191.1 | contig | Bacteria;Actinobacteria;Actinomycetia;Streptosporangiales;Thermomonosporaceae;Actinomadura |  |
| *Actinomadura sediminis* | JCM 31202 | *Actinomadura sediminis* JCM 31202 | JBHTJA01 | scaffold | Actinobacteria;Actinomycetia;Streptosporangiales;Thermomonosporaceae;Actinomadura |  |
| *Actinomadura sediminis* | YIM M 10931 | *Actinomadura sediminis* YIM M 10931 | JF272484 | contig | Bacteria;Actinobacteria;Actinomycetia;Streptosporangiales;Thermomonosporaceae;Actinomadura |  |
| *Actinomadura soli* | 14C53 | *Actinomadura soli* 14C53 | KX928704 | contig | Bacteria;Actinobacteria;Actinomycetia;Streptosporangiales;Thermomonosporaceae;Actinomadura |  |
| *Actinomadura sp.* | CC 0580 | *Actinomadura sp.* CC 0580 | FR744927.1 | contig | Bacteria;Actinobacteria;Actinomycetia;Streptosporangiales;Thermomonosporaceae;Actinomadura |  |
| *Actinomadura sp.* | CP26-28 | *Actinomadura sp.* CP26-28 | MT491108.1 | contig | Bacteria;Actinobacteria;Actinomycetia;Streptosporangiales;Thermomonosporaceae;Actinomadura |  |
| *Actinomadura sp.* | EGI 80046 | *Actinomadura sp*. EGI 80046 | KF040414.1 | contig | Bacteria;Actinobacteria;Actinomycetia;Streptosporangiales;Thermomonosporaceae;Actinomadura |  |
| *Actinomadura sp.* | SBMs009 | *Actinomadura sp*. SBMs009 | HQ883989.1 | contig | Bacteria;Actinobacteria;Actinomycetia;Streptosporangiales;Thermomonosporaceae;Actinomadura |  |
| *Actinomadura sp.* | 6K520 | *Actinomadura sp*. 6K520 | MG770874.1 | contig | Bacteria;Actinobacteria;Actinomycetia;Streptosporangiales;Thermomonosporaceae;Actinomadura |  |
| *Actinomadura sp.* | GC306 | *Actinomadura sp.* GC306 | MG770881.1 | contig | Bacteria;Actinobacteria;Actinomycetia;Streptosporangiales;Thermomonosporaceae;Actinomadura |  |
| *Actinomadura sp.* | 7K534 | *Actinomadura sp.* 7K534 | MK156410.1 | contig | Bacteria;Actinobacteria;Actinomycetia;Streptosporangiales;Thermomonosporaceae;Actinomadura |  |
| *Actinomadura sp.* | NAK00032 | *Actinomadura sp.* NAK00032 | CP054932.1 | chromosome | Bacteria;Actinobacteria;Actinomycetia;Streptosporangiales;Thermomonosporaceae;Actinomadura |  |
| *Actinomadura sp.* | 7K507 | *Actinomadura sp.* 7K507 | MG770779.1 | contig | Bacteria;Actinobacteria;Actinomycetia;Streptosporangiales;Thermomonosporaceae;Actinomadura |  |
| *Actinomadura sp.* | DHV1-7Ê | *Actinomadura sp*. DHV1-7Ê | MN058222.1 | contig | Bacteria;Actinobacteria;Actinomycetia;Streptosporangiales;Thermomonosporaceae;Actinomadura |  |
| *Actinomadura sp.* | DDPA2-13 | *Actinomadura sp.* DDPA2-13 | MN058271.1 | contig | Bacteria;Actinobacteria;Actinomycetia;Streptosporangiales;Thermomonosporaceae;Actinomadura |  |
| *Actinomadura sp.* | DDPA3-9 | *Actinomadura sp.* DDPA3-9 | MN058262.1 | contig | Bacteria;Actinobacteria;Actinomycetia;Streptosporangiales;Thermomonosporaceae;Actinomadura |  |
| *Actinomadura sp.* | 6N118 | *Actinomadura sp.* 6N118 | PP758764.1 | contig | Bacteria;Actinobacteria;Actinomycetia;Streptosporangiales;Thermomonosporaceae;Actinomadura |  |
| *Actinomadura spongiicola* | LHW52907 | *Actinomadura spongiicola* LHW52907 | MG200146 | contig | Bacteria;Actinobacteria;Actinomycetia;Streptosporangiales;Thermomonosporaceae;Actinomadura |  |
| *Actinomadura sporangiiformans* | NEAU-Jh2-5 | *Actinomadura sporangiiformans* NEAU-Jh2-5 | KM000834 | contig | Bacteria;Actinobacteria;Actinomycetia;Streptosporangiales;Thermomonosporaceae;Actinomadura |  |
| *Actinomadura sputi* | DSM 45233 | *Actinomadura sputi* DSM 45233 | FM957483 | contig | Bacteria;Actinobacteria;Actinomycetia;Streptosporangiales;Thermomonosporaceae;Actinomadura |  |
| *Actinomadura syzygii* | GKU 157 | *Actinomadura syzygii* GKU 157 | KF667496 | contig | Bacteria;Actinobacteria;Actinomycetia;Streptosporangiales;Thermomonosporaceae;Actinomadura |  |
| *Actinomadura terrae* | OS3-83 | *Actinomadura terrae* OS3-83 | FN178437 | contig | Bacteria;Actinobacteria;Actinomycetia;Streptosporangiales;Thermomonosporaceae;Actinomadura |  |
| *Actinomadura verrucosospora* | JCM 3147Ê | *Actinomadura verrucosospora* JCM 3147Ê | BAABEV01 | scaffold | Bacteria;Actinobacteria;Actinomycetia;Streptosporangiales;Thermomonosporaceae;Actinomadura |  |
| *Actinomadura verrucosospora* | NBRC 14100 | *Actinomadura verrucosospora* NBRC 14100 | U49011 | contig | Bacteria;Actinobacteria;Actinomycetia;Streptosporangiales;Thermomonosporaceae;Actinomadura |  |
| *Actinomadura vinacea* | JCM 3325 | *Actinomadura vinacea* JCM 3325 | AF134070 | contig | Bacteria;Actinobacteria;Actinomycetia;Streptosporangiales;Thermomonosporaceae;Actinomadura |  |
| *Actinomadura violacea* | LCR2-06 | *Actinomadura violacea* LCR2-06 | LC554835 | contig | Bacteria;Actinobacteria;Actinomycetia;Streptosporangiales;Thermomonosporaceae;Actinomadura |  |
| *Actinomadura viridis* | JCM 3112 | *Actinomadura viridis* JCM 3112 | BAABES01 | scaffold | Bacteria;Actinobacteria;Actinomycetia;Streptosporangiales;Thermomonosporaceae;Actinomadura |  |
| *Actinomadura viridis* | IFO 15238 | *Actinomadura viridis* IFO 15238 | D85467 | contig | Bacteria;Actinobacteria;Actinomycetia;Streptosporangiales;Thermomonosporaceae;Actinomadura |  |
| *Actinomadura xylanilytica* | KACC 20219 | *Actinomadura xylanilytica* KACC 20219 | JASNWE01 | scaffold | Bacteria;Actinobacteria;Actinomycetia;Streptosporangiales;Thermomonosporaceae;Actinomadura |  |
| *Actinomadura xylanilytica* | BK147 | *Actinomadura xylanilytica* BK147 | FR692101 | contig | Bacteria;Actinobacteria;Actinomycetia;Streptosporangiales;Thermomonosporaceae;Actinomadura |  |
| *Actinomadura yumaensis* | JCM 3369 | *Actinomadura yumaensis* JCM 3369 | AF163122 | contig | Bacteria;Actinobacteria;Actinomycetia;Streptosporangiales;Thermomonosporaceae;Actinomadura |  |
| *Spirillospora rubra* | JCM 6875 | *Spirillospora rubra* JCM 6875 | AF163123 | contig | Bacteria;Actinobacteria;Actinomycetia;Streptosporangiales;Thermomonosporaceae;Spirillospora |  |
| *Spirillospora sp.* | NBC_01491 | *Spirillospora sp.* NBC_01491 | CP109432.1 | chromosome | Bacteria;Actinobacteria;Actinomycetia;Streptosporangiales;Thermomonosporaceae;Spirillospora |  |
| *Spirillospora sp.* | NBC_00431 | *Spirillospora sp.* NBC_00431 | CP107927.1 | chromosome | Bacteria;Actinobacteria;Actinomycetia;Streptosporangiales;Thermomonosporaceae;Spirillospora |  |
| *Spirillospora tritici* | SJ 21 | *Spirillospora tritici* SJ 21 | MG271810 | contig | Bacteria;Actinobacteria;Actinomycetia;Streptosporangiales;Thermomonosporaceae;Spirillospora |  |
| *Thermomonospora umbrina* | JCM 6837 | *Thermomonospora umbrina* JCM 6837 | AF163121 | contig | Bacteria;Actinobacteria;Actinomycetia;Streptosporangiales;Thermomonosporaceae;Thermomonospora |  |
| *Thermomonospora umbrina* | DSM 43927 | *Thermomonospora umbrina* DSM 43927 | GCF_003386555.1 | contig | Bacteria;Actinobacteria;Actinomycetia;Streptosporangiales;Thermomonosporaceae;Thermomonospora |  |
| *Thermomonosporaceae sp.* | CNR431 | *Thermomonosporaceae sp*. CNR431 | AY464548.1 | contig | Bacteria;Actinobacteria;Actinomycetia;Streptosporangiales;Thermomonosporacea |  |
