# Supplementary material for: Actinomadura welshii sp. nov., a New Mycetoma Agent in Mexico
Source: PLoS Negl Trop Dis. 2025 Apr 11;19(4):e0013016. doi: 10.1371/journal.pntd.0013016 (PMC12021271; doi:10.1371/journal.pntd.0013016)
Supplement: S2 Table — (DOCX) [file pntd.0013016.s004.docx]

**S2 Table: Pairwise genome comparison of *LIID-AQ337* with the species of the GTDB database.** The table includes Genome ID, species name, ANI (Average Nucleotide Identity), and AF (Alignment Fraction), which indicate the genomic similarity between the query and reference genomes.

| **Genome id** | **Species** | **Radius (%)** | **ANI (%)** | **Alignment Fraction** |
| --- | --- | --- | --- | --- |
| GCF014648675.1 | *Spirillospora livida* | 95 | 89.87 | 0.576 |
| GCF004348665.1 | *Spirillospora sp004348665* | 95 | 89.86 | 0.568 |
| GCF004349245.1 | *Spirillospora sp004349245* | 95 | 89.81 | 0.561 |
| GCF006716765.1 | *Spirillospora hallensis* | 95 | 88.69 | 0.58 |
| GCF004348515.1 | *Spirillospora sp004348515* | 95 | 88.42 | 0.469 |
| GCF004348425.1 | *Spirillospora sp004348425* | 95 | 88.3 | 0.517 |
| GCF004348335.1 | *Spirillospora bangladeshensis* | 95 | 86.22 | 0.362 |
| GCF013372625.1 | *Spirillospora sp013372625* | 95 | 86.12 | 0.346 |
| GCF004348535.1 | *Spirillospora sp004348535* | 95 | 86.08 | 0.299 |
| GCF005889715.1 | *Spirillospora soli* | 95 | 86.02 | 0.306 |
| GCF001552155.1 | *Spirillospora formosensis* | 95 | 86.01 | 0.332 |
| GCF001552195.1 | *Spirillospora latina* | 95 | 85.94 | 0.38 |
| GCF005889745.1 | *Spirillospora geliboluensis* | 95 | 85.88 | 0.325 |
| GCF004348735.1 | *Spirillospora sp004348735* | 95 | 85.83 | 0.325 |
| GCF013364275.1 | *Spirillospora sp013364275* | 95 | 85.81 | 0.361 |
| GCF004349235.1 | *Spirillospora darangshiensis* | 95 | 85.51 | 0.346 |
| GCF900188445.1 | *Spirillospora meyerae* | 95 | 85.4 | 0.348 |
| GCF013409045.1 | *Spirillospora citrea* | 95 | 85.37 | 0.336 |
| GCF013409365.1 | *Spirillospora luteofluorescens* | 95 | 85.33 | 0.336 |
| GCF900115095.1 | *Spirillospora madurae* | 95 | 85.24 | 0.375 |
| GCF014208105.1 | *Spirillospora coerulea* | 95 | 85.21 | 0.328 |
| GCF006547145.1 | *Spirillospora sp006547145* | 95 | 85.2 | 0.325 |
| GCF900188105.1 | *Spirillospora mexicana* | 95 | 84.96 | 0.329 |
| GCF008327685.1 | *Spirillospora sp008327685* | 95 | 84.76 | 0.342 |
| GCF001552135.1 | *Spirillospora chibensis* | 95 | 84.59 | 0.291 |
| GCF003432485.1 | *Spirillospora spongiicola* | 95 | 84.35 | 0.252 |
| GCF008085905.1 | *Spirillospora syzygii* | 95 | 84.26 | 0.278 |
| GCF003634705.1 | *Spirillospora pelletieri* | 95 | 84.2 | 0.25 |
| GCF004349215.1 | *Spirillospora rubrisoli* | 95 | 84.14 | 0.216 |
| GCF008923205.2 | *Spirillospora physcomitrii* | 95 | 83.81 | 0.248 |
| GCF008923365.1 | *Spirillospora montaniterrae* | 95 | 83.65 | 0.26 |
| GCF030283205.1 | *Spirillospora sp030283205* | 95 | 83.53 | 0.257 |
| GCF900659615.1 | *Spirillospora fibrosa* | 95 | 83.38 | 0.221 |
| GCF017573545.1 | *Spirillospora nitritigenes* | 95 | 83.34 | 0.258 |
| GCF014706005.1 | *Spirillospora sp014706005* | 95 | 83.3 | 0.254 |
| GCF017573465.1 | *Spirillospora violacea* | 95 | 83.29 | 0.252 |
| GCF000718255.1 | *Spirillospora albida* | 95 | 83.15 | 0.226 |
| GCF014873935.1 | *Spirillospora algeriensis* | 95 | 83.03 | 0.202 |
| GCA014648495.1 | *Spirillospora cremea* | 95 | 82.98 | 0.211 |
| GCF022751215.1 | *Spirillospora terrae* | 95 | 82.97 | 0.176 |
| GCF019175365.1 | *Spirillospora graeca* | 95 | 82.94 | 0.191 |
| GCF009733595.1 | *Spirillospora litoris* | 95 | 82.93 | 0.185 |
| GCF000425065.1 | *Spirillospora rifamycini* | 95 | 82.82 | 0.202 |
| GCF008824145.1 | *Spirillospora sp008824145* | 95 | 82.81 | 0.194 |
| GCF001942465.1 | *Spirillospora sp001942465* | 95 | 82.78 | 0.202 |
| GCF900659635.1 | *Spirillospora roseirufa* | 95 | 82.78 | 0.168 |
| GCF001552215.1 | *Spirillospora macra* | 95 | 82.54 | 0.196 |
