# Supplementary material for: Actinomadura welshii sp. nov., a New Mycetoma Agent in Mexico
Source: PLoS Negl Trop Dis. 2025 Apr 11;19(4):e0013016. doi: 10.1371/journal.pntd.0013016 (PMC12021271; doi:10.1371/journal.pntd.0013016)
Supplement: S1 Fig — A, A. madurae LIID-AQ397; B, seven months after therapy with SXT alone. C Actinomadura LIID-AQ337; D, seven months after therapy with SXT plus amoxicillin/clavulanate. E, A. madurae LIID-AT157, F, decrease of lesions after therapy with 10 months of SXT and four cycles of amikacin. (DOCX) [file pntd.0013016.s005.docx]

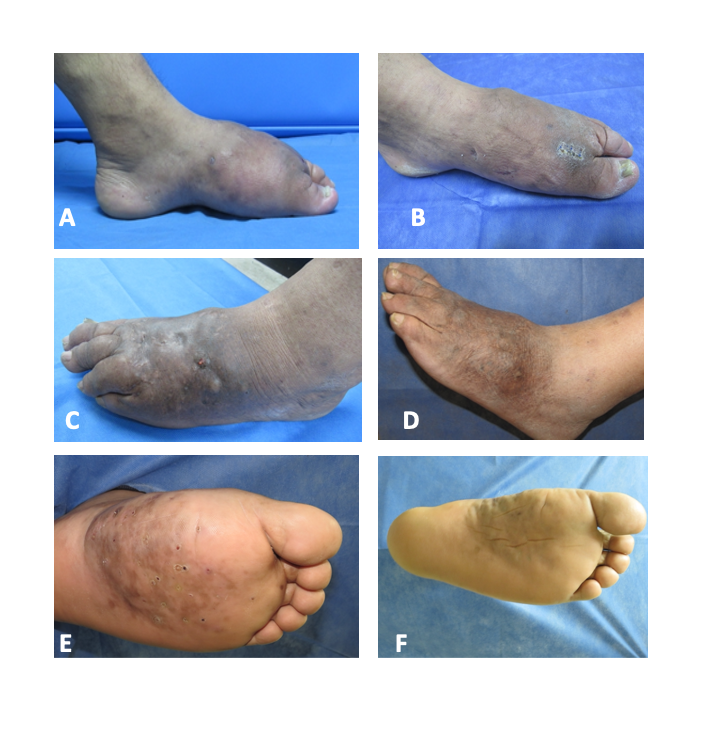
**S1 Fig. Clinical evolution of three of the mycetoma cases included in this study after therapy.** A, *A. madurae* LIID-AQ397; B, seven months after therapy with SXT alone. C *Actinomadura* LIID-AQ337; D, seven months after therapy with SXT plus amoxicillin/clavulanate. E, A*. madurae* LIID-AT157, F, decrease of lesions after therapy with 10 months of SXT and four cycles of amikacin.
