# Supplementary material for: Actinomadura welshii sp. nov., a New Mycetoma Agent in Mexico
Source: PLoS Negl Trop Dis. 2025 Apr 11;19(4):e0013016. doi: 10.1371/journal.pntd.0013016 (PMC12021271; doi:10.1371/journal.pntd.0013016)
Supplement: S2 Fig — In A we show the presence of granules > 1 mm size (10x) in the pus of patient LIID-AT157. In B we show a 40x magnification to show the presence of filaments of less 1 μm diameter. In C we show the presence of typical large basophilic grains in an H&E stained tissue. Bar represents 200 μm. (DOCX) [file pntd.0013016.s006.docx]

**
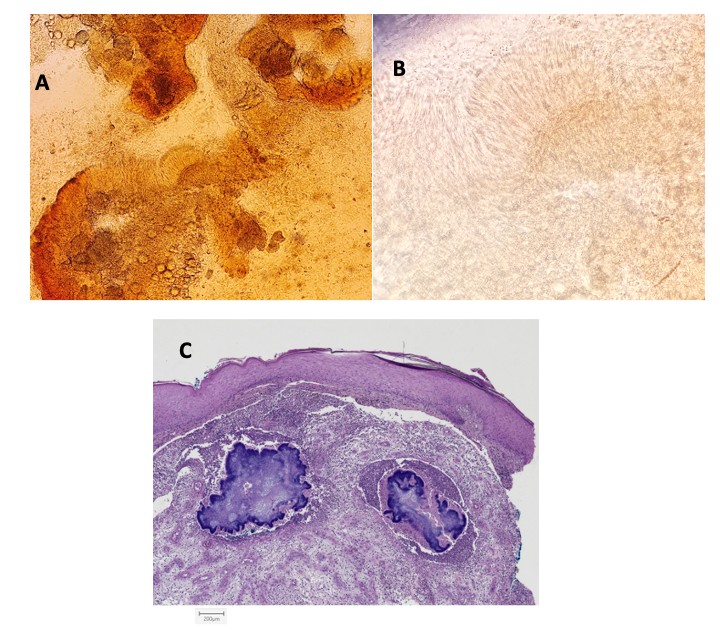
S2 Fig. Microscopic examination**. In A we show the presence of granules > 1 mm size (10x) in the pus of patient LIID-AT157. In B we show a 40x magnification to show the presence of filaments of less 1 μm diameter. In C we show the presence of typical large basophilic grains in an H&E stained tissue. Bar represents 200 μm.
