# Supplementary material for: Actinomadura welshii sp. nov., a New Mycetoma Agent in Mexico
Source: PLoS Negl Trop Dis. 2025 Apr 11;19(4):e0013016. doi: 10.1371/journal.pntd.0013016 (PMC12021271; doi:10.1371/journal.pntd.0013016)
Supplement: S3 Fig — The alignment was done using Geneious Prime v2024-0–7. This fragment aligned with the nucleotide sequence (positions 3058936–3058745) of the genome sequence of A. madurae DSM43067 (GenBank accession no. X97889) encoding for the 16S ribosomal RNA product. (DOCX) [file pntd.0013016.s007.docx]

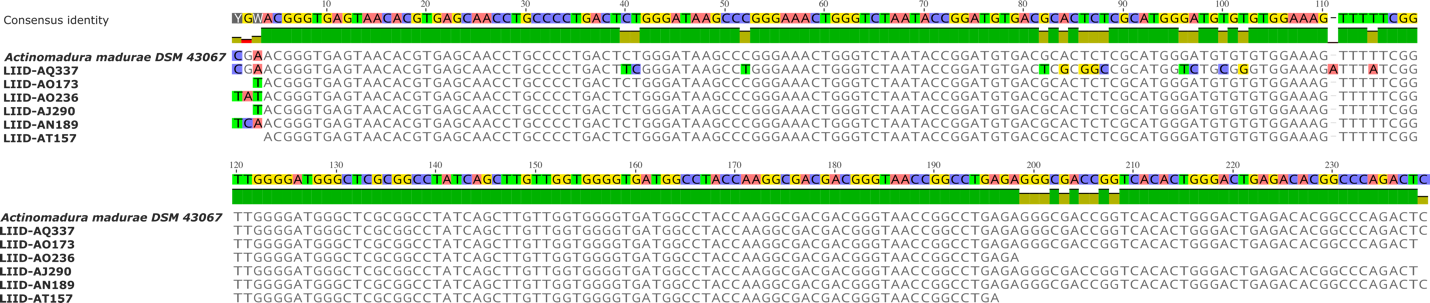


**S3 Fig. Nucleotide sequence alignment of the 16S rRNA amplicons (NOC3-NOC4) derived from the *Actinomadura* isolates from this study and DSM43067 as reference.** The alignment was done using Geneious Prime v2024-0-7. This fragment aligned with the nucleotide sequence (positions 3058936 to 3058745) of the genome sequence of *A. madurae* DSM43067 (GenBank accession no. X97889) encoding for the 16S ribosomal RNA product.
