# Supplementary material for: Actinomadura welshii sp. nov., a New Mycetoma Agent in Mexico
Source: PLoS Negl Trop Dis. 2025 Apr 11;19(4):e0013016. doi: 10.1371/journal.pntd.0013016 (PMC12021271; doi:10.1371/journal.pntd.0013016)
Supplement: S4 Fig — Inferred using MEGA11 with a bootstrap test of phylogeny with 1000 replicates and a site coverage cutoff of 90%. The closest species is Actinomadura hibisca NBRC15177 with a bootstrap 100. (DOCX) [file pntd.0013016.s008.docx]

# **
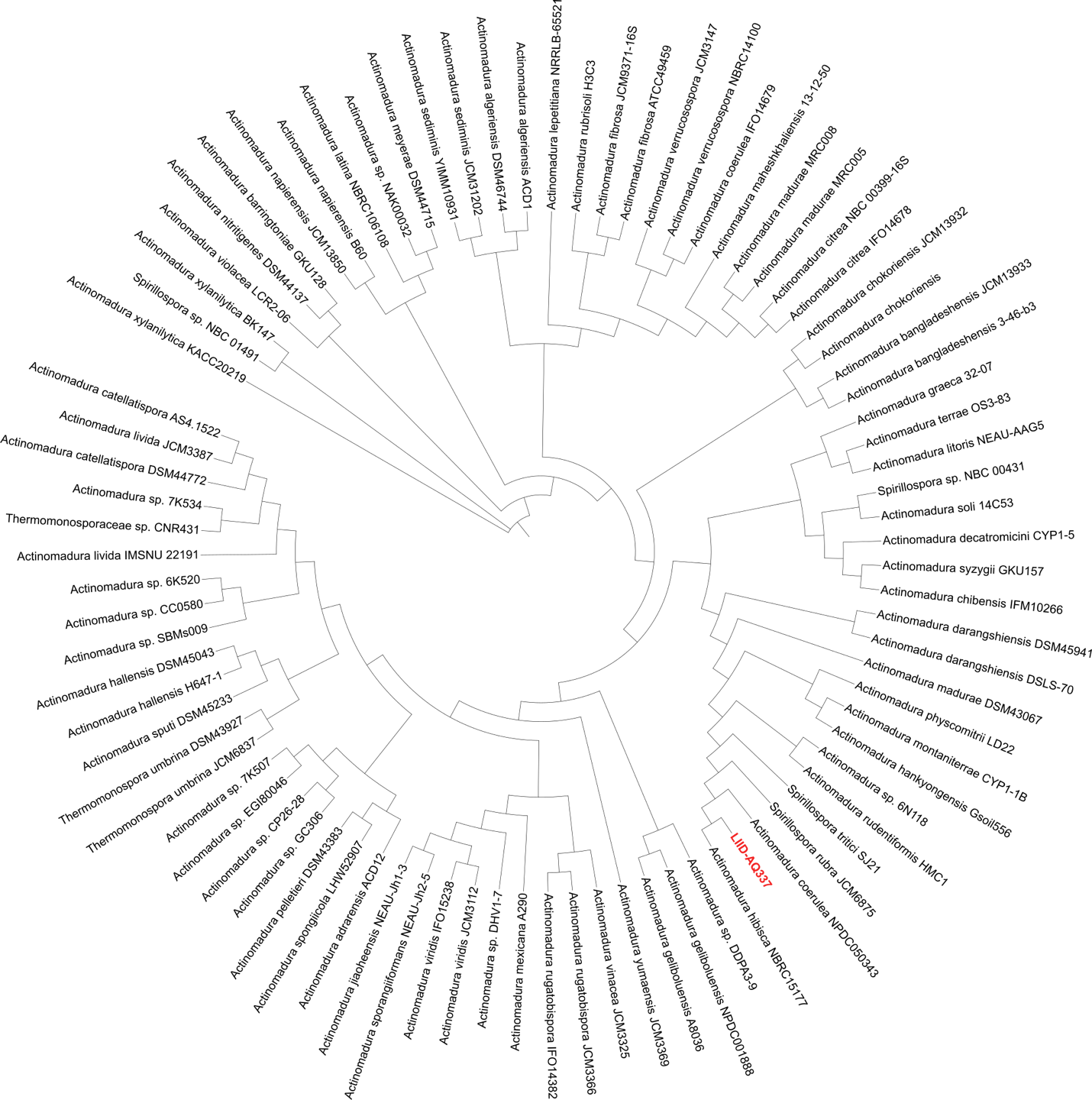
**

# **S4 Fig. Maximum parsimony tree of the 16S rRNA phylogeny of Actinomadura** showing the placement of LIID-AQ337 (red) with related 16S rRNA sequences from the EZBioCloud and NCBI. Inferred using MEGA11 with a bootstrap test of phylogeny with 1000 replicates and a site coverage cutoff of 90%. The closest species is *Actinomadura hibisca* NBRC15177 with a bootstrap 100.
